# Vaccination With Oral Polio Vaccine Reduces COVID-19 Incidence

**DOI:** 10.3389/fimmu.2022.907341

**Published:** 2022-05-30

**Authors:** Nadezhda V. Yagovkina, Lev M. Zheleznov, Ksenia A. Subbotina, Andrey A. Tsaan, Liubov I. Kozlovskaya, Ilya V. Gordeychuk, Anastasia K. Korduban, Yury Y. Ivin, Anastasia A. Kovpak, Anastasia N. Piniaeva, Anna A. Shishova, Elena Y. Shustova, Yusuf K. Khapchaev, Galina G. Karganova, Alexandra A. Siniugina, Tatiana V. Pomaskina, Aleksandr A. Erovichenkov, Konstantin Chumakov, Aydar A. Ishmukhametov

**Affiliations:** ^1^ Center for Clinical Trials, Kirov State Medical University, Russian Ministry of Health, Kirov, Russia; ^2^ Department of Epidemiology, Perm State Medical University, Ministry of Health, Perm, Russia; ^3^ RIC-Pharma, Moscow, Russia; ^4^ Chumakov Federal Scientific Center for Research and Development of Immune-and-Biological Products of Russian Academy of Sciences, Global Virus Network Center of Excellence, Moscow, Russia; ^5^ Institute of Translational Medicine and Biotechnology, Sechenov First Moscow State Medical University, Moscow, Russia; ^6^ Biopolis-Kirov 200 Subsidiary of Chumakov Center for Research and Development of Immunobiological Products, Kirov, Russia; ^7^ Department of Infectious Diseases, Russian Medical Academy of Continuous Professional Education of the Ministry of Health, Moscow, Russia; ^8^ U.S. Food and Drug Administraion (FDA) Office of Vaccines Research and Review, Global Virus Network Center of Excellence, Silver Spring, MD, United States

**Keywords:** SARS-CoV-2, innate immunity, non-specific protection, vaccine off-target effects, emerging diseases

## Abstract

**Background:**

Effective response to emerging pandemic threats is complicated by the need to develop specific vaccines and other medical products. The availability of broadly specific countermeasures that could be deployed early in the pandemic could significantly alter its course and save countless lives. Live attenuated vaccines (LAVs) were shown to induce non-specific protection against a broad spectrum of off-target pathogens by stimulating innate immune responses. The purpose of this study was to evaluate the effect of immunization with bivalent Oral Poliovirus Vaccine (bOPV) on the incidence of COVID-19 and other acute respiratory infections (ARIs).

**Methods and Findings:**

A randomized parallel-group comparative study was conducted in Kirov Medical University. 1115 healthy volunteers aged 18 to 65 were randomized into two equal groups, one of which was immunized orally with a single dose of bOPV “BiVac Polio” and another with placebo. The study participants were monitored for three months for respiratory illnesses including COVID-19. The endpoint was the incidence of acute respiratory infections and laboratory confirmed COVID-19 in both groups during 3 months after immunization. The number of laboratory-confirmed cases of COVID-19 was significantly lower in the vaccinated group than in placebo (25 cases vs. 44, p=0.036). The difference between the overall number of clinically diagnosed respiratory illnesses in the two groups was not statistically significant.

**Conclusions:**

Immunization with bOPV reduced the number of laboratory-confirmed COVID-19 cases, consistent with the original hypothesis that LAVs induce non-specific protection against off-target infections. The findings are in line with previous observations of the protective effects of OPV against seasonal influenza and other viral and bacterial pathogens. The absence of a statistically significant effect on the total number of ARIs may be due to the insufficient number of participants and heterogeneous etiology of ARIs. OPV could be used to complement specific coronavirus vaccines, especially in regions of the world where the vaccines are unavailable, and as a stopgap measure for urgent response to future emerging infections. Clinical trial registration number NCT05083039 at clinicaltrals.gov https://clinicaltrials.gov/ct2/show/NCT05083039?term=NCT05083039&draw=2&rank=1

## Introduction

The first case of the novel coronavirus infection (COVID-19) caused by a virus named Severe Acute Respiratory Syndrome Coronavirus 2 (SARS-CoV-2) was detected at the end of 2019 in the city of Wuhan in Hubei province of China. The main manifestations of the disease were atypical pneumonia with high mortality, especially in the elderly and people with preexisting conditions (1). The virus quickly spread throughout the world causing one of the biggest pandemics in recent history.

During an emerging pandemic, rapid interventions are of paramount importance to slow the pathogen transmission until protective vaccines are developed and introduced. In early phases of COVID-19 pandemic methods for preventing and the spread SARS-CoV-2 included epidemiological measures such as physical distancing, use of personal protective equipment, contact tracing, restrictions on the size of meetings or business activities, as well as travel restrictions and quarantines (2, 3). These measures result in the disruption of both social and economic activities, which makes finding the right balance between them critical to minimizing their negative impact (4). Although such restrictions are highly effective, they can only be a temporary measure and the ultimate solution must involve establishment of population immunity that would prevent transmission of the virus to stop the pandemic.

Development of specific vaccines against a new pathogen is a lengthy and challenging process that normally takes years. Close cooperation between governments, industry, and academia that was made possible because of the urgency created by the pandemic, resulted in first vaccines released under emergency use authorizations and even fully licensed in a record time. Nevertheless, this unprecedented success is tempered by the challenges in the worldwide rollout of the vaccines, especially in low-income countries with insufficient public health infrastructure. Another challenge is the emergence of new virus variants with reduced sensitivity to vaccine-induced immunity. If the antigenic drift continues, this may require continuous revaccinations with modified vaccines, as in the case of influenza. Therefore, availability of universal broadly protective vaccines would be highly desirable. Their deployment early in pandemic cycle could limit the spread of the new pathogen and even prevent a local outbreak from becoming a global pandemic.

Many live attenuated vaccines (LAVs) were shown to induce broad protection not only against their target pathogens, but also against other unrelated infections (5–7). This was shown for Bacillus Calmette-Guerin (BCG, vaccine against tuberculosis) (8, 9), live vaccine against measles (10), Oral Poliovirus Vaccine (OPV) (11), and some other vaccines (12). Their introduction was found to reduce the all-cause mortality in low-income countries (13, 14) and infectious disease-related hospitalizations in high-income countries (15). A prospective clinical study conducted in the Soviet Union in the late 1960s demonstrated that OPV could reduce the incidence of influenza and other acute respiratory diseases (16, 17). OPV use in Bangladesh was found to reduce the burden of bacterial diarrhea (18). In other studies, OPV vaccination was shown to prevent otitis media (19) and promote healing of genital herpes lesions (17). The mechanism of this broadly protective effects of LAVs involves stimulation of innate immunity that serves as the first line of defense against infecting pathogen. Therefore, it has been proposed that LAVs could be used as a broadly effective tool for preventing COVID-19 (20, 21).

One of the most attractive LAVs is OPV. It is by far the least expensive vaccine (few cents per dose), it is administered orally, and is highly safe for fully immunized individuals. There are no direct studies demonstrating protective effects of OPV vaccination against COVID-19. However, comparison between countries using OPV and those that use only inactivated polio vaccine (IPV) showed that the former have a lower incidence of COVID-19 (22). In addition, a recent study demonstrated that mothers indirectly exposed to attenuated poliovirus after their babies were immunized with OPV had a much lower incidence of COVID-19 (23). Here we report the results of a clinical study of the effect of OPV immunization of adults on the incidence of COVID-19 and other respiratory infections. The study was conducted in the Kirov Region of Russia and demonstrated that OPV reduced the number of confirmed COVID-19 cases 1.8-fold. Further studies are urgently needed to reveal the precise mechanism and the duration of this protection and to identify the best way of using these broad protective effects of OPV.

## Methods

### Study Design and Participants

The study was designed as a single-center, prospective, randomized, parallel-group comparative study conducted at the Kirov Medical University between May 14, 2020 and January 11, 2021. The volunteers were recruited in the city of Kirov and Kirov Region. The enrolment was conducted at several local enterprises and among general population.

### Enrollment and Randomization

**Figure 1:**
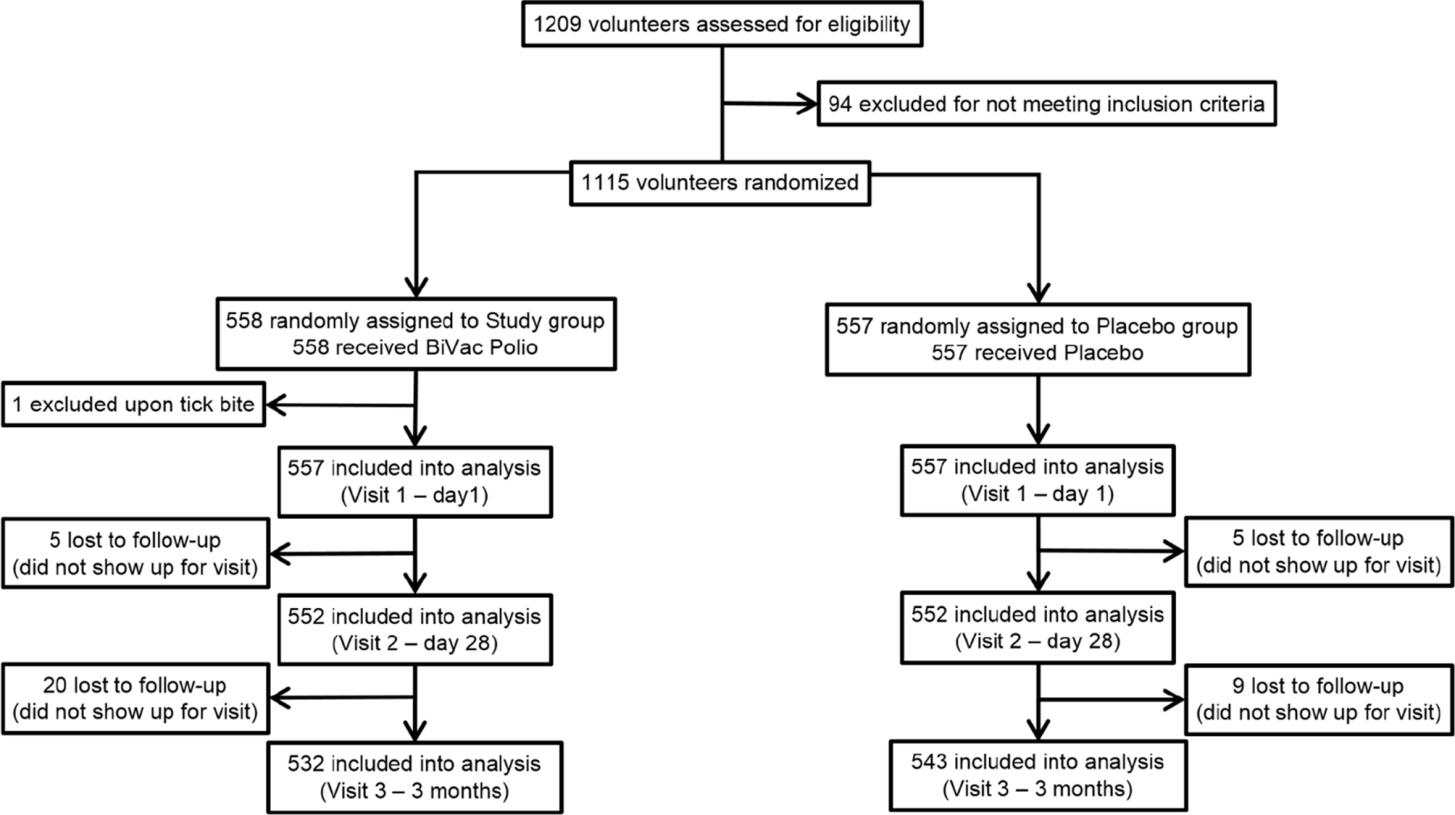
Flow Diagram of the study.

During the screening stage, 1209 male and female volunteers between the ages of 18 and 65 were considered for enrollment into the study (**Figure 1**). The inclusion criteria were good general health (defined as the absence of chronic conditions and abnormalities identified during the collection of medical anamnesis and standard clinical examination), written and dated informed consent to participate in the study and follow the protocol, consent to take precautions to limit the circulation of the vaccine virus among contact persons (personal hygiene, isolation from unvaccinated children and persons with immunodeficiencies). The exclusion criteria were previous infection with SARS-CoV-2 (including asymptomatic cases), positive PCR test for COVID-19 at enrollment or administration of other vaccines one month prior to screening, neurological disorders following the previous OPV vaccinations, primary immunodeficiency, malignant neoplasms, immunosuppression, pregnancy, hypersensitivity to any component of the vaccine, fever (temperature above 40°C) or other complications to the previous administration, acute infectious or non-infectious diseases. Persons whose family members or contacts were not vaccinated against poliomyelitis (e.g., newborns or children with contraindications to polio immunization) or were immunodeficient were excluded from the study. Based on the examination results, assessment of inclusion and exclusion criteria, 1115 volunteers, 645 women and 470 men (57.8 and 42.2%, respectively) were randomized between the study and placebo groups. During the selection, all volunteers were subjected to nasopharyngeal swab PCR test for SARS-CoV-2 RNA presence, medical anamnesis collection, physical examination, and an assessment of neurological status.

All enrolled participants were randomized into 2 groups using interactive system IWRS. The groups were comparable by age, sex, body weight, height, and body mass index (BMI) (**Table 1**).

**Table 1:** Average demographic and anthropometric characteristics of study participants.

Parameter	Statistics	Group		Total	Group comparison
		bOPV (BiVac Polio) (N=558)	Placebo (N=557)	(N=1115)	(t-test, р-value)
Age, years	Average ± SD	42.2 ± 12.2	41.6 ± 12.5	41.9 ± 12.3	t =0.565, p = 0.935
Sex	Male/Female	241/317	229/328	470/645	t = 0.702, p = 0.483
Body mass, kg	Average ± SD	75.1 ± 17.3	75.5 ± 15.9	75.3 ± 16.7	t =0.475, p = 0.635
Height, cm	Average ± SD	169.9 ± 9.8	169.8 ± 9.7	169.8 ± 9.7	t = 0.383, p = 0.702
BMI, kg/m^2^	Average ± SD	25.9 ± 5.1	26.1 ± 4.8	26.0 ± 5.0	t = 1.149, p = 0.251

### Procedures

The study group (n = 558) received a single dose (0.2 ml) of bivalent OPV types 1 and 3 (“BiVac polio”), the participants of the placebo group (n = 557) received a placebo (distilled sterilized water in the same volume).

**Figure 2:**
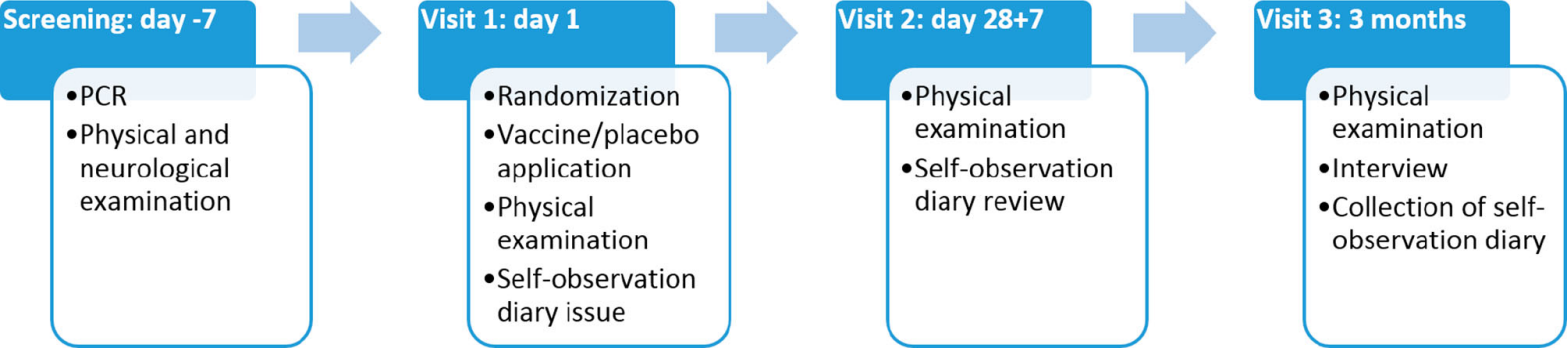
Timeline of the study.

Throughout the duration of the study participants kept self-observation diaries, returned for three visits (**Figure 2**), and were contacted by telephone to monitor their health. Any undesirable event reported by the participant was recorded throughout the entire study. At the final visit, each participant was asked to answer a questionnaire regarding possible contacts with persons with symptoms of acute respiratory infections, as well as with confirmed COVID-19 diagnosis. Participants who dropped out of the study were not replaced. The overall study scheme is presented in **Figure 1**.

Blood was collected on each visit from 20 participants in placebo group and from 40 participants in vaccinated group, chosen randomly. Poliovirus neutralizing antibodies (NAb) in the sera were determined in microneutralization assay in НEр-2c cells using Sabin strains of types 1 and 3 according to the standard WHO protocol (24).

### Outcomes

The primary endpoint of the study was the number of detected cases of COVID-19, laboratory confirmed by PCR and/or serological tests, within 3 months, by group.

The secondary endpoints were: (i) The total number of cases of acute respiratory infections (e.g. influenza, common cold, etc.) in groups, and for laboratory-confirmed cases of COVID-19, and (ii) the severity of the disease; the duration of illness; the presence, nature and severity of complications; the number of hospitalizations; the number of lethal outcomes (none in either group).

### Statistical Analysis

The disease incidence and other parameters in the groups (frequency analysis) were compared using the Chi-square test and t-test. In addition, risk ratio and odds ratios were calculated to assess the risk of COVID-19 occurrence between groups. Calculations were performed using IBM SPSS Statistics 23 and the free open-source computing environment within the framework of the GNU project (R version 4.1.0).

## Results

### Study Population

Study participants in both groups were people with stable family relationships (**Table 2**) on average having two children in each family. The groups did not differ in their marital status. The professional composition of the groups was also comparable. As expected, medical workers (25.4% in the vaccinated and 24.4% in placebo groups, respectively) were most interested in participating in the study. Participants in labor occupations accounted for 18.5% and 17.0%, respectively, teachers of various educational institutions in Kirov and the Kirov region - 4.7% and 4.8%, executives of various levels – 3.8% and 4.5%, respectively. Students of secondary specialized and higher education institutions were represented at 6.9% in study group and 9.5% in placebo group. Unemployed comprised 5.8% and 3.9%, respectively. Thus, the groups were similar in terms of demographic and social structure.

**Table 2:** Socio-demographic characteristics of study participants.

Social factor		bOPV (BiVac Polio) (n=557)	Placebo (n=557)
Marital status	Marriage (official and unofficial)	80.0 ± 2.9	78.7 ± 3.0
Children	One child	41.0 ± 4.4	42.3 ± 4.4
	Two or more children	20.0 ± 2.9	17.0 ± 2.5
Occupation	Health professional	25.4 ± 3.4	24.4 ± 3.3
	Industrial workers	18.5 ± 2.7	17.0 ± 2.5
	Teacher	4.7 ± 0.8	4.8 ± 0.8
	Executive	3.8 ± 0.7	4.5 ± 0.8
	Manager	5.1 ± 0.9	5.0 ± 0.2
	Student	6.9 ± 1.2	9.5 ± 1.5
	Engineer	5.1 ± 0.9	7.2 ± 1.2
	Unemployed	5.8 ± 1.0	3.9 ± 0.7
	Other	23.6 ± 3.3	22.6 ± 3.1

The screening questionnaire included questions on previous history of acute respiratory infections (ARI) (**Table 3**). Analysis of the previous incidence of ARI in both groups revealed that most often the participants were sick twice a year (58.3% in study group and 57.9% in placebo group), or once a year (21.4% and 22.2% respectively). The proportion of frequently ill patients was 13.2 in study group and 13.1 in placebo group. 86.6% of the participants in study group and 85.5% in placebo group were aware of their previous vaccination with poliomyelitis vaccine. The data agrees with the official information on the implementation of vaccination within the framework of the National Vaccination Schedule in Russia. Thus, the overall health status of participants in both groups also appeared to be similar.

**Table 3:** Incidence of prior acute respiratory infections (ARI) and polio vaccination status among study participants based on questionnaire responses.

Parameter		bOPV (BiVac Polio)	Placebo
		(n=557)	(n=557)
ARI	1 time a year	21.4 ± 1.7%	22.2 ± 1.8%
	2 times a year	58.3 ± 2.1%	57.9 ± 2.1%
	> 4 times a year	13.2 ± 1.4%	13.1 ± 1.4%
Previous polio vaccination (tOPV)		86.6 ± 1.4%	85.5 ± 1.5%

None of the participants was vaccinated against COVID-19 prior to the enrollment or during the study, as the COVID-19 vaccines were not available at that time.

### Immunization with OPV

Since all participants were previously immunized against poliomyelitis, it was important to demonstrate that they can successfully respond to vaccination with OPV. Sera samples were collected from a subset of participants in both groups on visits 1 and 2 and tested for neutralization activity against attenuated Sabin polioviruses. At the beginning of the study all participants had detectable antibodies against at least two poliovirus serotypes; 2 out of 20 in placebo and 6 out of 40 in the study group did not have detectable NAb titers (<1:8) against type 3 poliovirus, which may indicate waning immunity against this serotype.

One month after vaccination (visit 2) NAb titers in the placebo group did not increase, the average difference between pre- and post-placebo titers was zero. At the same time in vaccinated group neutralization titers increased on average 5.6-fold against type 1 poliovirus, and 16-fold against type 3 poliovirus. All immunized participants seroconverted, demonstrating that OPV-vaccinated participants have successfully responded to the administration of the vaccine despite the prior immunity to poliovirus.

The information about cases of COVID-19 and ARI was collected during scheduled visits (on day 28 and after 3 months), by telephone calls from study participants who became ill, self-observation diary (data collected on each visit) as well as by questionnaire completed during the final visit. Some study participants did not inform the study staff about their illness at the time of diagnosis and reported the case only during the visits, providing the results of a positive PCR test for SARS-CoV-2 RNA or a written diagnosis from the hospital where they were treated. Therefore, the final analysis of the study results included the overall incidence of respiratory infections and COVID-19 among the participants.

### Incidence of COVID-19

Analysis of the number of confirmed COVID-19 cases demonstrated that the difference between OPV-vaccinated and placebo groups was statistically significant: 25 vs. 44 cases (44.9 per 1000 vs. 79.0 per 1000; p=0.036) (**Table 3**). Thus, in the group that received OPV, the incidence of COVID-19 was 1.8 times lower than in placebo group, suggesting the protective efficacy of OPV against COVID-19. No deaths were observed in either group.

In agreement with this data, assessment of the odds ratio showed that among patients with COVID-19, the chance of identifying people who did not receive OPV was 1.8 times higher than among those vaccinated with OPV (OR = 1.8 [1.10; 3.03]).

Additional analysis of COVID-19 cases that occurred within one month after the administration of OPV showed that in the vaccinated group there were 4 confirmed cases, compared to 7 in the placebo group. This difference may suggest that (i) the effect of OPV administration is evident almost immediately, and (ii) it is not limited to the first month after vaccination. However, because of the small number of cases that occurred in the first month the difference did not reach the level of statistical significance. Estimation of the relative risks showed that the risk of contracting COVID-19 during the first month in the group receiving placebo was 1.9 times higher than in the group of participants vaccinated with OPV. Similar results were obtained for the odds ratio: among patients with COVID-19, the chance of identifying individuals not vaccinated with OPV was 2.3 times higher than among vaccinated subjects (OR = 2.3 [0.60; 8.69]). However, because of the small number of cases observed in this study as indicated above, the question about the longevity of the protection remains unresolved.

### Incidence of Other Acute Respiratory Infections

A total of 68 cases of self-reported ARI (**Tables 3**, **4**) were registered in the study group versus 66 cases in placebo group. Analysis that included only laboratory-confirmed cases showed that the incidence of ARI was somewhat lower in the vaccinated group than in placebo group (107.7 vs. 114.9 per 1000, respectively), but the difference was not statistically significant.

**Table 4:** Cases of COVID-19 and other acute respiratory illnesses in bOPV recipient and placebo groups.

Incidence of	bOPV (BiVac Polio)		Placebo		Statistical analysis	
	(n=557)		(n=557)			
	1 month	3 months	1 month	3 months	1 month	3 months
Acute respiratory infections, total ^a^	28	68	29	66	χ^2 =^ 0.000, p=1.000	χ^2 =^ 0.005, p=0.943
	(5.03 ± 0.9%)	(12.2 ± 1.4%)	(5.2 ± 0.9%)	(11.8 ± 1.4%)		
Acute respiratory infections, lab-confirmed	20	60	19	64	χ^2 =^ 0.000, p=1.000	χ^2 =^ 0.060, p=0.806
	(3.4 ± 0.8%)	(10.8 ± 1.2%)	(3.4 ± 0.8%)	(11.5 ± 1.2%)		
COVID-19, lab-confirmed ^b^	4	25	7	44	χ2 = 0.2, p=0.655	χ^2 **=**^ **4.393, p=0.036** ^c^
	(0.7 ± 0.4%)	(4.5 ± 1.2%)	(1.3 ± 0.5%)	(7.9 ± 1.2%)		

a Self-reported (questionnaire) and lab-confirmed (medical records).

b PCR-positive.

c Difference is statistically significant, p<0.05 (shown in Bold).

## Discussion

The results presented in this communication showed that the incidence of COVID-19 was lower in the group that received OPV compared to placebo. Similarly, the risk of contracting COVID-19 was lower among OPV recipients that in placebo group. The frequency of a positive PCR test or the presence of serum antibodies to SARS-CoV-2 among participants who had contacts with persons with signs of ARI or diagnosed with COVID-19 was significantly higher in the placebo group than among vaccinated. These observations suggest that vaccination with OPV may confer increased resistance to coronavirus infection, consistent with similar observations made in the late 1960s during the Influenza season (16, 17). The level of protection induced by OPV against COVID-19 was also consistent with earlier observations made for influenza and is comparable with the efficacy of some antigen-specific coronavirus vaccines.

It is noteworthy that while being immunized with OPV years prior, all vaccinated participants successfully responded to OPV by increased serum neutralizing antibody levels. This suggests that OPV can be administered repeatedly. An important advantage of OPV is that it exists in three serotypes, allowing each monovalent vaccine to be used sequentially, expanding the duration of cross-protection against COVID-19 that is most likely mediated by stimulation of interferon production and activation of other innate immunity pathways. This stimulation is expected to be transient, and it is important to determine the duration of this effect. The results presented here showed that in the first month there were four COVID-19 cases among OPV recipients and seven in the placebo group. Even though the difference did not reach the level of statistical significance because of the small number of cases, it was comparable to the overall reduction of COVID-19 cases during the entire observation period of three months. This suggests that the broadly protective effect of OPV lasts for more than one month. Additional studies are needed to determine the duration of this protection more accurately. It is also important to determine whether OPV can induce a more long-lasting protection through the trained immunity mechanisms that were demonstrated for other LAVs (25–27).

Some observations (e.g., the effect of OPV on the number of ARI cases) were not statistically significant, probably because of the small number of participants in the study and heterogeneous etiology of ARI. This emphasizes the need for larger follow-up studies to establish the magnitude and the duration of the protection, and the detailed biological mechanisms behind it.

Although this study provides the initial direct evidence for a substantial effect of OPV against cases of COVID-19, it does have several limitations. These include:

- The study only assessed COVID-19 cases, not infections. Because of limited availability of PCR tests, they were used only to confirm COVID-19 diagnosis. In the future studies it will be desirable to assess all infections, including asymptomatic, e.g., by testing for antibodies to internal proteins of SARS-CoV2.
- Similarly, this study included too few subjects to be able to assess the effect of OPV on mortality or severe disease, possibly because the study did not include high-risk individuals with pre-existing conditions, such as diabetics, obese, and elderly. Given the general finding that vaccines against COVID-19 are more effective against severe disease than against milder cases, it would be important — when sufficiently large numbers of subjects are available — to evaluate this dimension of impact.
- This study was not designed or powered to address several important follow-on questions about differential effects across different subgroups such as age or time of enrollment with its potential implications for incidence during the time of the subject’s enrollment. As the previous literature has found important gender differences in response to LAVs this aspect of impact is also worth assessing. For this, larger or longer lasting trials would be required.
- Finally, as has been noted above the use of OPV in practice is very likely to be in conjunction with COVID-19 vaccines when and where they are available rather than as a substitute for them. The existing trial provides critical information on OPV administered before the COVID-19 specific vaccine becomes available but is unable to answer the question of the effect of simultaneous administration. Will effects be additive or synergistic? It can be hypothesized that stimulation of innate immunity by OPV could provide an adjuvating effect on antigen specific COVID-19 vaccines. This possibility must be explored in a separate study. However, we emphasize that the obvious use of heterologous LAVs is at the outset of any new epidemic caused by a virus for which there is no vaccine, as well as in regions of the world where specific vaccines are unavailable.

The demonstration of protective efficacy of OPV against COVID-19 confirms multiple other observational studies that revealed off-target effects of LAVs (28) and supports earlier proposals to use OPV and other LAVs for emergency protection against COVID-19 (21). Despite that there are several new vaccines against COVID-19, we believe that there is a place for OPV and other LAVs as a complimentary tool to control the pandemic, especially in low-income countries struggling with effective roll-out of relatively expensive new vaccines.

Off-target protection induced by LAVs could be used for rapid response to emerging diseases. If deployed early in the pandemic cycle, they could change its course and provide protection during the period when specific vaccines are being developed and rolled out. Another important advantage of using off-target effects of LAVs is their broad specificity suggesting that their efficacy will not be affected by the emerging virus variants with altered antigenicity.

OPV is a very inexpensive vaccine, it is easy to administer and very safe. However, it can cause very rare complications in persons with primary immunodeficiency (29). Due to its genetic instability, it can generate circulating vaccine-derived polioviruses (cVDPV, primarily of serotype 2) in communities with insufficient population immunity (30). Recently a novel genetically stable strain of OPV2 was created and is currently used under WHO Emergency Use Listing to control outbreaks of cVDPV2 (31, 32). Similar novel strains of nOPV1 and nOPV3 are in advanced phases of preclinical development. It is expected that use of these strains will reduce or eliminate risks associated with conventional OPV use, making them also attractive as a broadly protective countermeasure against emerging infections.

An important question is how to harness the broadly protective effects if OPV and other LAVs in the public health context. OPV is currently used to immunize children in more than 140 countries, requiring close to 1 billion doses each year. Its expanded use to control emerging diseases would disrupt supply of the vaccine for its intended purpose. Ramping up production of the vaccine may take several months; however, frozen bulks of the vaccine are highly stable and could be prepared in advance and stockpiled for emergency use. In case of an emergence of a new infection the vaccine could be rapidly used for ring vaccination to contain the outbreak or for mass vaccination as a stopgap measure to protect the population until antigen-specific vaccines are developed and rolled out.

In conclusion, we believe that studies of the broadly protective effects of OPV and other LAVs must be urgently expanded to determine their detailed mechanism and the best way they should be used, because they may be a currently missing universal tool for the preparedness to future pandemics.

## Data Availability Statement

The original contributions presented in the study are included in the article/supplementary material. Further inquiries can be directed to the corresponding authors.

## Ethics Statement

The studies involving human participants were reviewed and approved by Center for clinical trials, Kirov State Medical University, Russian Ministry of Health. The patients/participants provided their written informed consent to participate in this study.

## Author Contributions

NY, Study design, data analysis, manuscript preparation. LZ, Study design and coordination, manuscript preparation. KS, Study design, Data analysis. AT, Study design and coordination. LK, Study design, laboratory analyses, manuscript preparation. IG, Data analysis. AKK, Study coordination. YI, Laboratory analyses. AAK, Laboratory analyses. AP, Laboratory analyses. AnS, Laboratory analyses. ES, Laboratory analyses. YK, Study Coordination. GK, Study design. AAS, Study Coordination. TP, Study Coordination. AE, Data analysis. KC, Study Idea, Manuscript preparation. AI, Study design and coordination. All authors contributed to the article and approved the submitted version.

## Funding

The study was performed using intramural funding of participating organizations.

## Conflict of Interest

The authors declare that the research was conducted in the absence of any commercial or financial relationships that could be construed as a potential conflict of interest. Chumakov Federal Scientific Center for Research and Development of Immune-and-Biological Products of RAS is the manufacturer of “BiVac Polio” bivalent Oral Poliovirus Vaccine. This did not affect the conduct of the study.

## Publisher’s Note

All claims expressed in this article are solely those of the authors and do not necessarily represent those of their affiliated organizations, or those of the publisher, the editors and the reviewers. Any product that may be evaluated in this article, or claim that may be made by its manufacturer, is not guaranteed or endorsed by the publisher.
